# Mammographic density by time and breast: a retrospective cohort study from BreastScreen Norway

**DOI:** 10.1186/s13058-025-02037-2

**Published:** 2025-05-16

**Authors:** Nataliia Moshina, Jonas Gjesvik, Tone Hovda, Henrik W. Koch, Heinrich A. Backmann, Solveig Hofvind

**Affiliations:** 1 The Cancer Registry of Norway, Department of Screening programs, Norwegian Institute of Public Health, Oslo, Norway; 2 Department of Radiology, Vestre Viken Hospital Trust, Drammen, Norway; 3 Department of Radiology, Stavanger University Hospital, Stavanger, Norway; 4 Faculty of Health Sciences, University of Stavanger, Stavanger, Norway; 5 Department of Radiology, Nordland Hospital Trust, Bodø, Norway; 6 Department of Health and Care Sciences, Faculty of Health Sciences, UiT The Arctic University of Norway, Tromsø, Norway

**Keywords:** Breast cancer, Risk, Breast density, Mammography, Screening

## Abstract

**Background:**

Mammographic density is known to decrease over time in postmenopausal women. Longitudinal changes in mammographic density prior to breast cancer diagnosis have been widely discussed and less density reduction has been observed for breast developing versus not developing cancer. We aimed to verify these findings among participants of BreastScreen Norway.

**Methods:**

In this retrospective cohort study, data from 78,182 women aged 50–69 years who attended three consecutive screening rounds between 2007 and 2020 were included. Among those women, 970 were diagnosed with screen-detected and 308 with interval cancer. Mammographic density data was obtained from an automated software and included absolute (cm^3^) and percent (%) dense volume for each breast and for each woman, per examination. A linear mixed-effects regression model estimating differences in density between the breast developing and not developing cancer was applied to evaluate longitudinal changes, separately for absolute and percent dense volume. The model was adjusted for age at first screening examination, breast volume, follow-up time, history of benign breast disease, body mass index, family history, hormone therapy, use of alcohol and smoking. Results were presented as linear regression coefficient estimates with 95% confidence intervals (CI).

**Results:**

Mean age at the third screening examination for women without breast cancer was 62.5 (standard deviation, SD: 5.1) years, while mean age at diagnosis was 62.3 (SD: 4.4) years for women with screen-detected cancer and 61.9 (SD: 4.8) years for women with interval cancer. In our model, absolute and percent dense volume decreased with follow-up time, estimate=-0.010 (95%CI -0.010; -0.009) and estimate=-0.013 (95%CI -0.014; -0.013), respectively, indicating the overall negative effect of time on mammographic density. The interaction between time and development of breast cancer was positive for absolute and percent dense volume, estimate = 0.009 (95%CI 0.004; 0.014) for both, which implied that mammographic density in breasts developing cancer was stable or slightly decreasing.

**Conclusions:**

Less reduction in longitudinally assessed mammographic density was observed for breasts developing versus not developing cancer in our study. This difference might be used for more precise 4–6 years breast cancer risk prediction and screening personalization.

**Supplementary Information:**

The online version contains supplementary material available at 10.1186/s13058-025-02037-2.

## Background

Mammographic density is an independent risk factor for breast cancer [1]. In addition, dense breast tissue may mask tumors on mammograms, resulting in a higher risk of advanced tumors in women with high versus low density [2]. The U.S. has introduced the law on breast density notification as a result of screening mammography, and supplemental screening methods are offered for women with dense breasts [3, 4]. In Europe, the practice of notification is less common, but supplemental screening methods for women with extremely dense breasts have been recommended by the European Society of Breast Imaging (EUSOBI) since 2022 [5–7]. Mammographic density and breast parenchymal patterns were first described by Wolfe in 1974 [8] and Tabar in 1982 [9]. The American College of Radiology introduced the Breast Imaging Reporting and Data System (BI-RADS) in 1992, including a 4-point classification system for mammographic density [10, 11]. The BI-RADS system has been revised, and the fifth version still implies four categories: (a) The breasts are almost entirely fatty, (b) There are scattered areas of fibroglandular density, (c) The breasts are heterogeneously dense, which may obscure small masses, (d) The breasts are extremely dense, which lowers the sensitivity of mammography [12]. However, persistent inter-reader variability of subjective density assessment has led to the introduction of automated methods [13]. Several semi- and fully automated systems for density measurement have been developed and CE- and FDA approved [14, 15]. Various types of fully automated software for mammographic density assessment offer the possibility to objectively estimate the percent dense volume (volumetric breast density), absolute dense volume (fibroglandular volume), and the total volume of the breast [16, 17]. The positive association of percent and absolute dense volume with breast cancer risk has been shown in several studies [1, 15, 18].

In an epidemiological context, evidence on changes in mammographic breast density over time has been extensively described, demonstrating that mammographic density decreases by age and is associated with body mass index (BMI) and breast volume [19–22]. However, less is known about the individual woman’s longitudinal changes in mammographic density and possible differences between the two breasts. The results from the few studies are inconsistent [23, 24], and the issue has not been investigated prospectively. Such knowledge could be of substantial importance in the discussion and implementation of personalized breast cancer screening. Furthermore, adding mammographic density to breast cancer risk score provided by artificial intelligence (AI) has been shown to increase the accuracy of the AI systems [25, 26]. A recent study from the U.S. showed that percent dense volume decreased to a lower extent over time in breasts developing versus not developing breast cancer in a multiethnic cohort of women screened 2008–2020 [23]. The study was limited by number of cases and data from non-population-based screening facilities.

In this study, we intended to address the aspects described. We used data collected as a part of BreastScreen Norway, an organized population-based breast cancer screening program for women aged 50–69, including individual information on mammographic density measured by an automated system, screening information, including cancer detection, and risk factors for breast cancer [17, 27]. The automated system provided continuous density measurements for each breast and an overall density measurement for each woman per examination [17, 28].

We aimed to analyze changes in mammographic density over three consecutive screening rounds among women developing and not developing breast cancer, on a breast level and on an individual level.

## Methods

This study had a legal basis in accordance with Articles 6 [1] (e) and 9 [2] (a) of the GDPR [29]. The data was disclosed with a legal basis in the Cancer Registry Regulations Sects. 3 − 1 and the Personal Health Filing System Act Sect. 19 a to 19 h [30]. The use of data was approved by the Regional Committee for Medical and Health Research Ethics (510838).

### Study sample

Data was extracted from the Cancer Registry of Norway, which administers BreastScreen Norway. The screening program started in 1996 and offers biennial mammography to about 680,000 women aged 50–69 [27]. Over the last 10 years, about 84% of invited women have attended the program at least once [27]. A screening examination includes two-view mammography: left and right craniocaudal (CC) and mediolateral oblique (MLO). Solely information from CC view was included in the study, as one view (either CC, MLO, or an average value for CC and MLO) has previously been shown to be sufficient to assess association between percent dense volume and breast cancer and predict future breast cancer risk [31].

**Fig. 1:**
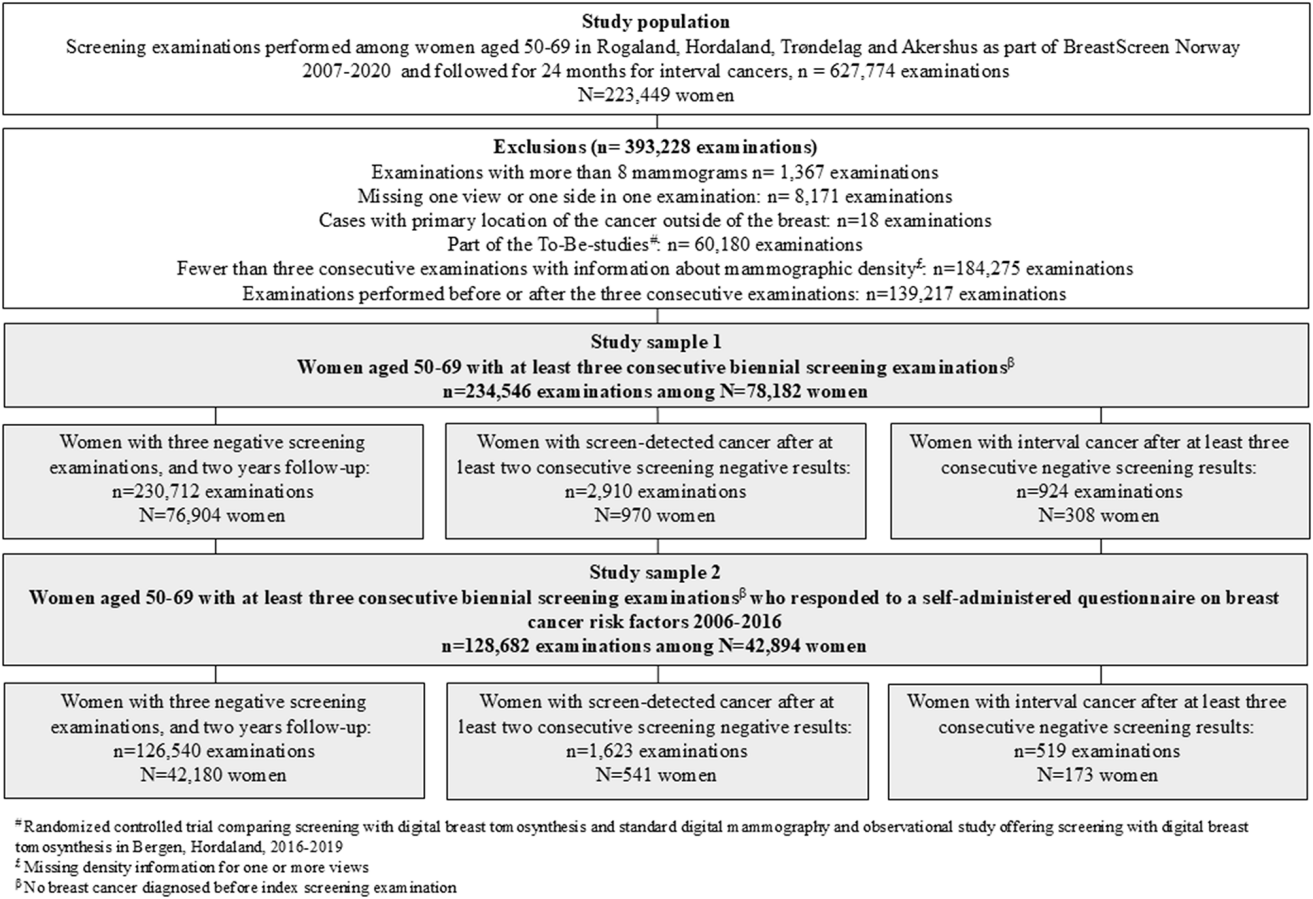
Study samples with exclusions

Information about women screened in the Norwegian counties Rogaland, Hordaland, Trøndelag and Akershus during the period January 1, 2007 – December 31, 2020, were included in the study (Fig. 1). In this period, data on mammographic density measured by an automated system (Volpara, versions 1.5.0 and 1.5.4.0) was collected [17]. Information on risk factors was collected with a self-administered questionnaire among women screened in the period from 2006 to 2016 [32].

We excluded examinations with more than eight mammograms (*n* = 1367) and those missing one view or one side in one examination (*n* = 8171). Further, examinations of women diagnosed with a primary cancer other than breast cancer (*n* = 18 examinations), those who participated in the To-Be-studies (*n* = 60,180 examinations) [33], and those who participated in fewer than three consecutive examinations (*n* = 184,275 examinations) were excluded. Finally, we excluded examinations performed before or after the three consecutive examinations included in the study (*n* = 139,217 examinations). Due to these exclusions, women with bilateral breast cancers were not a part of the study sample. The exclusions left 234,546 examinations among 78,182 women for Study sample 1. Study sample 2 included data from a subset of women who responded to a questionnaire on risk factors related to breast cancer 2006–2016 (128,682 examinations from 42,894 women, Table 1). Both study samples were further stratified into three groups: (1) women with three negative screening examinations, and a two-year follow-up (Study sample 1: 230,712 examinations in 76,904 women; Study sample 2: 126,540 examinations in 42,180 women), (2) women with screen-detected cancer after at least two consecutive examinations with negative results (Study sample 1: 2910 examinations in 970 women; Study sample 2: 1623 examinations in 541 women) and (3) women with at least three consecutive screening examinations with negative screening results and diagnosed with interval cancer within two years after the last screening examination (Study sample 1: 924 examinations in 308 women; Study sample 2: 519 examinations in 173 women).

**Table 1:** Mean age at third examination or diagnosis, time of follow-up and distribution of breast cancer risk factors for women with or without screen-detected or interval cancer among participants of BreastScreen Norway, 2007–2020

Variable	Women with screen-detected breast cancer	Women with interval breast cancer	Women with screen-detected and interval breast cancer	Women without breast cancer	Total
**Study sample 1**	*n* = 970	*n* = 308	*n* = 1278	*n* = 76,904	*n* = 78,182
Age (years, mean, SD, range)	62.2 (4.4, 53–71)	61.8 (4.8, 53–70)	62.1 (4.5, 53–71)	62.3 (5.0, 52–71)	62.3 (5.0, 52–71)
Time of follow-up (days, mean, SD)	1503.4 (53.5)	1943.8 (180.0)	1609.5 (213.3)	2217.4 (59.4)	2207.4 (100.8)
History of benign breast disease (n, %)	32 (3.3%)	8 (2.6%)	40 (3.1%)	1759 (2.3%)	1799 (2.3%)
Volpara Density Grade 5th edition					
1 (n, %)	120 (12.4%)	14 (4.5%)	134 (10.5%)	15,831 (20.6%)	15,965 (20.4%)
2 (n, %)	534 (55.1%)	136 (44.2%)	670 (52.4%)	39,937 (51.9%)	40,607 (51.9%)
3 (n, %)	273 (28.1%)	128 (41.6%)	401 (31.4%)	17,602 (22.9%)	18,003 (23.0%)
4 (n, %)	43 (4.4%)	30 (9.7%)	73 (5.7%)	3534 (4.6%)	3607 (4.6%)
**Study sample 2**	*n* = 541	*n* = 173	*n* = 714	*n* = 42,180	42,894
Age (years, mean, SD, range)	62.1 (4.3, 53–71)	61.3 (4.7, 53–70)	61.9 (4.4, 53–71)	62.4 (4.7, 52–71)	62.4 (4.7, 52–71)
Time of follow-up (days, mean, SD)	1500.6 (49.2)	1922.8 (192.2)	1602.9 (208.6)	2216.8 (58.1)	2206.6 (101.1)
History of benign breast disease (n, %)	16 (3.0%)	5 (2.9%)	21 (2.9%)	979 (2.3%)	1000 (2.3%)
Volpara Density Grade 5th edition					
1 (n, %)	60 (11.1%)	7 (4.2%)	67 (9.4%)	8661 (20.5%)	8728 (20.3%)
2 (n, %)	301 (55.6%)	70 (40.5%)	371 (52.0%)	21,920 (52.0%)	22,291 (52.0%)
3 (n, %)	156 (28.8%)	78 (45.1%)	234 (32.8%)	9726 (23.1%)	9960 (23.2%)
4 (n, %)	24 (4.4%)	18 (10.4%)	42 (5.9%)	1873 (4.4%)	1915 (4.5%)
Body mass index (kg/m^2^, mean, SD)	26.4 (5.2)	25.5 (4.2)	26.2 (5.0)	25.7 (4.3)	25.7 (4.4)
First- or second-degree family history of breast cancer, yes, n, %)	60 (11.1%)	24 (13.9%)	84 (11.8%)	4116 (9.8%)	4200 (9.8%)
Ever used hormone therapy in screening period (n, %)	244 (45.1%)	83 (48.0%)	327 (45.8%)	15,477 (36.7%)	15,804 (36.8%)
Ever used alcohol (n, %)	418 (77.3%)	136 (78.6%)	554 (77.6%)	33,248 (78.8%)	33,802 (78.8%)
Ever smoked (n, %)	304 (56.2%)	91 (52.6%)	395 (55.3%)	23,443 (55.6%)	23,838 (55.6%)

SD - standard deviation

**Table 2:** ABC. Mean values with standard deviation (SD) and median values with interquartile range (IQR) for (**A**) absolute dense volume; (**B**) breast volume; and (**C**) percent dense volume on an individual level for women developing and not developing screen-detected or interval breast cancer during three consecutive screening rounds of biennial screening in BreastScreen Norway, 2007–2020

A. Absolute dense volume (cm^3^)	Screening round		
	First	Second	Third
Women developing screen-detected cancer (*n* = 970)			
Mean (SD)	53.0 (25.0)	52.7 (25.1)	52.2 (24.8)
Median (IQR)	47.3 (35.2–64.3)	45.9 (35.2–65.7)	46.6 (35.0–63.0)
Women developing interval cancer (*n* = 308)			
Mean (SD)	59.6 (33.7)	59.1 (34.9)	59.2 (32.9)
Median (IQR)	50.1 (37.3–69.0)	49.1 (35.9–70.2)	50.7 (38.0–69.8)
Women developing screen-detected or interval cancer (*n* = 1278)			
Mean (SD)	54.6 (27.5)	54.2 (27.9)	53.9 (27.1)
Median (IQR)	48.2 (35.6–65.3)	46.4 (35.2–67.1)	47.4 (35.7–65.2)
Women not developing breast cancer (*n* = 76,904)			
Mean (SD)	47.7 (23.5)	46.9 (22.9)	45.8 (22.4)
Median (IQR)	42.1 (32.6–56.1)	41.5 (32.1–55.1)	40.5 (31.4–53.8)
B. Breast volume (cm^3^)	Screening round		
	First	Second	Third
Women developing screen-detected cancer (*n* = 970)			
Mean (SD)	904.8 (457.1)	919.6 (454.5)	936.2 (455.6)
Median (IQR)	829.5 (577.4–1150.4)	834.7 (597.3–1160.2)	857.9 (615.5–1193.2)
Women developing interval cancer (*n* = 308)			
Mean (SD)	795.0 (417.0)	815.3 (424.4)	823.9 (417.5)
Median (IQR)	700.9 (476.9–1047.9)	739.4 (514.5–1026.7)	745.7 (522.1–1083.3)
Women developing screen-detected or interval cancer (*n* = 1278)			
Mean (SD)	878.4 (450.0)	894.5 (449.5)	909.2 (449.1)
Median (IQR)	797.6 (551.4–1125.0)	814.1 (581.7–1134.7)	831.2 (587.8–1164.7)
Women not developing breast cancer (*n* = 76,904)			
Mean (SD)	879.0 (447.0)	897.1 (444.9)	914.2 (447.2)
Median (IQR)	809.6 (555.0–1130.2)	828.8 (577.9–1147.2)	850.8 (592.0–1166.2)
C. Percent dense volume (%)	Screening round		
	First	Second	Third
Women developing screen-detected cancer (*n* = 970)			
Mean (SD)	7.0 (4.0)	6.7 (3.7)	6.6 (3.7)
Median (IQR)	5.7 (4.2–8.6)	5.6 (4.2–8.2)	5.5 (4.0–8.0)
Women developing interval cancer (*n* = 308)			
Mean (SD)	8.9 (5.1)	8.5 (4.9)	8.3 (4.4)
Median (IQR)	7.3 (5.3–11.1)	7.2 (5.0–10.4)	7.3 (4.9–10.3)
Women developing screen-detected or interval cancer (*n* = 1278)			
Mean (SD)	7.4 (4.4)	7.1 (4.1)	7.0 (4.0)
Median (IQR)	6.1 (4.4–9.2)	5.9 (4.3–8.7)	5.8 (4.2–8.5)
Women not developing breast cancer (*n* = 76,904)			
Mean (SD)	6.6 (4.0)	6.3 (3.8)	6.1 (3.9)
Median (IQR)	5.3 (3.9–8.0)	5.1 (3.7–7.6)	4.8 (3.5–7.4)

### Variables of interest

The screening interval in BreastScreen Norway is 24 months +/-6 months, which means that the screening round is approximately 2 years [27]. Data on age (years) at screening, history of benign breast disease, screening round (1, 2 and 3 in the study period) and method of diagnosis were obtained from the Cancer Registry. Volpara^™^ was used to estimate absolute dense volume (cm^3^), breast volume (cm^3^), and percent dense volume (%) [17]. Volpara provided continuous density measurements for each breast and an overall density measurement for each woman per examination [17]. The algorithm and density estimation are described elsewhere [28]. The mammograms were classified into four Volpara density grades (VDG 1–4) based on the maximum percent dense volume for each examination [34]. This VDG classification was similar but not analogous to the BI-RADS fifth edition classification [11].

Data on weight and height at the time of the examination, first- or second-degree family history of breast cancer, use of hormone therapy after menopause (ever/never), use of alcohol (ever/never), and smoking (ever/never) were extracted from the questionnaire on risk factors [32]. The data from the most recent response to the questionnaire was used in the study. BMI (kg/m^2^) was calculated for each woman as weight divided by squared height based on data reported in the questionnaire.

Screen-detected breast cancer was defined as histologically verified breast cancer (ductal carcinoma in situ [DCIS] or invasive breast cancer) diagnosed after a positive screening examination within a 6-month period following the screening examination. Interval cancer was defined as histologically verified breast cancer detected within 24 months after a negative screening result or in the interval of 6–24 months after a false positive screening examination.

Further, we defined the breast where breast cancer was detected as “the breast developing cancer”, while the contralateral breast was defined as “the breast not developing cancer”. For women not developing cancer, the breasts were defined as “breasts without cancer”.

### Statistical analysis

Descriptive information included means with standard deviations (SD), medians and interquartile ranges (IQR) and/or range for the continuous variables and numbers with percentages for the categorical variables, for women with screen-detected and interval cancer and for women without breast cancer. Mean and median absolute dense volume, breast volume and percent dense volume for the same groups of women, as well as breasts developing and not developing screen-detected or interval cancers in women with cancer, were presented by screening round. A linear mixed-effects regression model was fitted to the data to evaluate longitudinal association between developing cancer and mammographic density presented as linear regression coefficient estimates with 95% confidence intervals (CI). We fitted the model on breast level with a dichotomous term defining if the breast developed cancer, and a separate term if the breast did not develop cancer, but the woman did. Breasts in women without cancer were used as reference [35]. We adjusted for age at first screening examination in this study, breast volume, follow-up time (screening rounds, 1, 2, and 3 with a 2-year interval between the rounds (screening interval), plus 2 years of follow-up for interval cancer), history of benign breast disease (grouped as ever/never diagnosed), BMI, first- or second-degree family history of breast cancer (versus no), ever use of hormone therapy after menopause (versus never), ever use of alcohol (versus never), and ever smoking (versus never), and added a random constant intercept on the level of each woman to adjust for individual differences. A separate term for possible interaction between follow-up time and developing cancer for each breast was used to evaluate the change in absolute dense volume over the three screening rounds [35]. An analogous set of analyses was conducted for percent dense volume. This regression model was also applied for absolute and percent dense volume with adjustment only for age at first screening examination, breast volume as a substitute for BMI, follow-up time, and history of benign breast disease using Study sample 1 (Additional file 1, Table S2). Spearman correlation coefficients were used to analyze associations between breast volume and BMI over the consecutive screening rounds. We fitted similar linear-mixed regression analyses on absolute or percent dense volume adjusting for the same confounders as in the main model on an individual level instead of breast level. Box-Cox transformation was used to normalize the distribution of absolute and percent dense volume, as the evaluation of their residuals showed a skewed distribution (Additional file 2, Figure S1-S2). Further analyses using missing values or tumor diameter as confounders did not result in any changes in the observed associations and were not included. Analyses were performed in Stata MP 18.5 (College Station, TX: StataCorp LLC).

## Results

Study sample 1 included information from 78,182 women with three consecutive screening examinations, a total of 234,546 examinations. Of these, 970 women were diagnosed with screen-detected breast cancer in the third screening round and 308 were diagnosed with interval cancer. Mean age for the women without breast cancer was 62.3 years (SD: 5.0), 62.2 (SD: 4.4) for women with screen-detected cancer, and 61.8 (SD: 4.8) for those with interval cancer (Table 1). Among women without breast cancer, 20.6% were classified with VDG1 (15,831/76,904), 51.9% with VDG2 (39,937/76,904), 22.9% with VDG3 (17,602/76,904) and 4.6% with VDG4 (3534/76,904) at the last screening round in the study. The distribution was 10.5% (134/1278) for VDG1, 52.4% (670/1278) for VDG2, 31.4% (401/1278) for VDG3 and 5.7% (73/1278) for VDG4 for women who developed screen-detected and interval cancer. Characteristics of women in Study sample 1 and 2 did not differ substantially (Table 1).

On an individual level, mean and median absolute dense volume was stable during three consecutive screening rounds in women diagnosed with screen-detected and interval cancer and decreased in women who did not develop breast cancer (Table 2A). Mean and median breast volume consistently increased over time resulting in decreasing percent dense volume in all three groups (Table 2B and C).

**Fig. 2:**
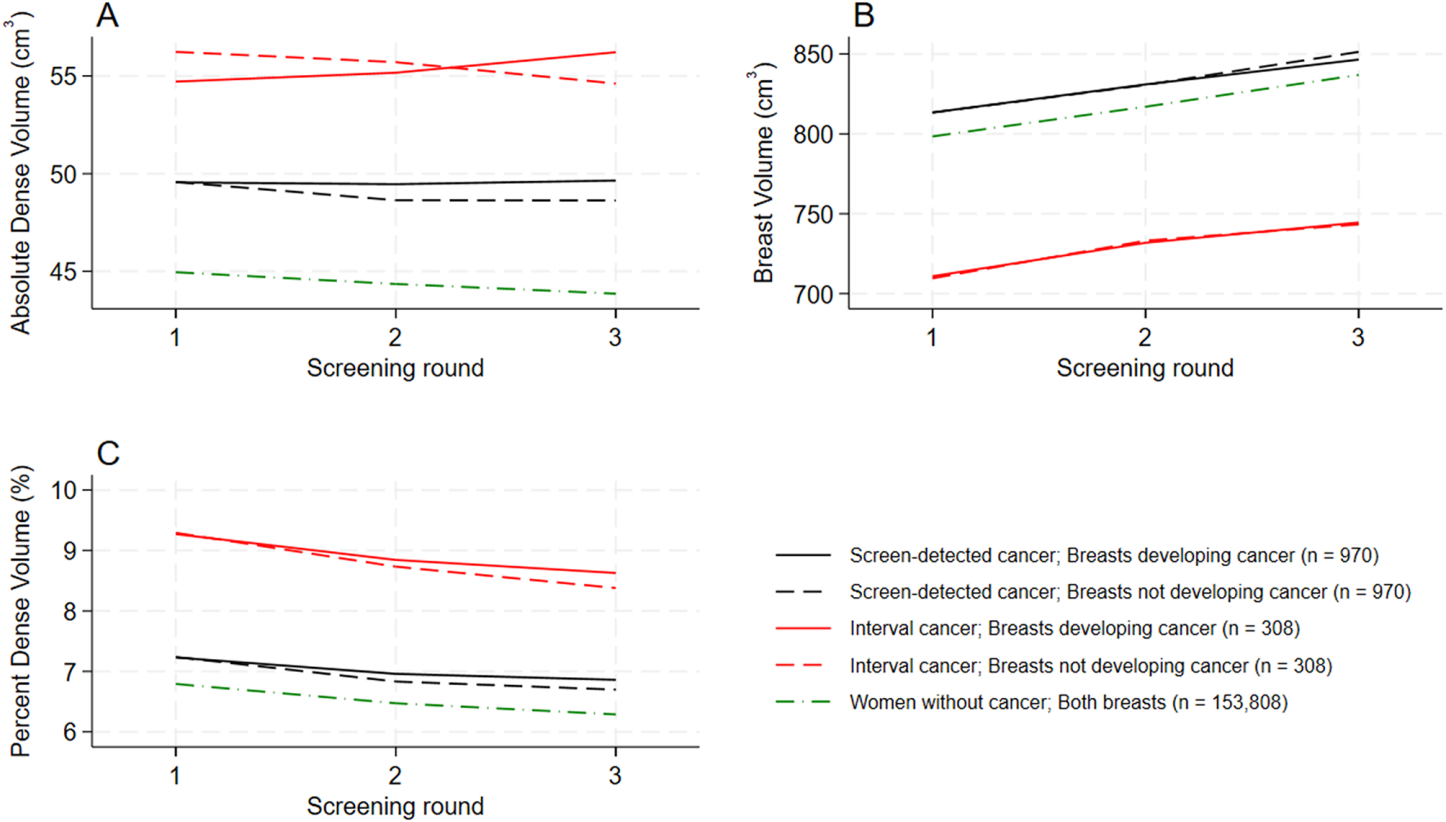
Means of (**A**) absolute dense volume (cm^3^), (**B**) breast volume (cm^3^) and (**C**) percent dense volume (%) for breasts developing or not developing screen-detected or interval breast cancer and women without breast cancer over three consecutive screening rounds in BreastScreen Norway, 2007–2020

On a breast level, absolute dense volume did not differ by time in breasts developing screen-detected cancer over three consecutive screening rounds but slightly increased in the breasts developing interval cancer (Fig. 2A and Additional file 1, Table S1). Both absolute and percent dense volume were lower, while breast volume was higher in women diagnosed with screen-detected cancer compared to women diagnosed with interval cancer, and women without breast cancer (Table 2A and Fig. 2).

In linear mixed-effects regression analyses on a breast level, absolute dense volume decreased with follow-up time for all women, estimate=-0.010 (95%CI -0.010; -0.009, *p* < 0.001), and was higher both for the breasts developing cancer (estimate = 0.056, 95%CI 0.038; 0.075, *p* < 0.001), and the breasts not developing cancer (estimate = 0.060, 95%CI 0.042; 0.079, *p* < 0.001) in women with breast cancer, using breasts without cancer as reference (Table 3). An analogous trend was observed for percent dense volume. Increasing BMI was associated with decreasing percent dense volume (estimate = -0.005, 95%CI -0.005; -0.004, *p* < 0.001). In the breasts developing cancer, the rate of the longitudinal decrease was lower for absolute (estimate = 0.009, 95%CI 0.004; 0.014, *p* < 0.001) and percent dense volume (estimate = 0.009, 95%CI 0.004; 0.014, *p* < 0.001) compared to breasts without cancer, in the models adjusted for age at first screening round, breast volume, benign breast disease, follow-up time (screening round), BMI, family history, hormone therapy, use of alcohol and smoking. Similar results were observed in a model using Study sample 1 (Additional file 1, Table S2) with breast volume as a substitute for BMI due to high correlation between the variables (Additional file 1, Table S3). In linear mixed-effects regression analyses on an individual level, women who developed cancer showed a lower decrease in absolute and percent dense volume compared to women without breast cancer, estimate = 0.006, 95%CI 0.001; 0.011, *p* = 0.027 for absolute dense volume, and estimate = 0.007, 95%CI 0.002; 0.012, *p* = 0.009 for percent dense volume (Additional file 1, Table S4).

**Table 3:** Estimates obtained from a linear-mixed regression model with 95% confidence intervals (CI) showing the change in absolute (cm^3^) and percent (%) dense volume in each breast over three consecutive screening rounds and association with breast cancer for 42,894 women screened in BreastScreen Norway, 2007–2020

Variable	Absolute dense volume (cm^3^) **			Percent dense volume (%) **		
	*n* = 128,682 examinations (*n* = 42,894 women)^§^			*n* = 128,682 examinations (*n* = 42,894 women)^§^		
	**Estimate (95% CI)**	**P-value**		**Estimate (95% CI)**	**P-value**	
Age at first screening examination (years)	-0.006 (-0.008; -0.005)	< 0.001		-0.007 (-0.008; -0.005)	< 0.001	
Breast volume (cm^3^)	0.0002 (0.0002; 0.0002)	< 0.001		-0.0004 (-0.0004; -0.0004)	< 0.001	
Benign breast disease (ever in BreastScreen Norway)	0.003 (-0.010; 0.016)	0.61		0.009 (-0.003; 0.021)	0.14	
Follow-up time (screening round)	-0.010 (-0.010; -0.009)	< 0.001		-0.013 (-0.014; -0.013)	< 0.001	
Breast not developing cancer	0.060 (0.042; 0.079)	< 0.001		0.063 (0.046; 0.080)	< 0.001	
Breast developing cancer	0.056 (0.038; 0.075)	< 0.001		0.058 (0.040; 0.075)	< 0.001	
Breasts without cancer^&^	Reference			Reference		
Follow-up time*breast not developing cancer	0.002 (-0.003; 0.007)	0.42		0.003 (-0.002; 0.007)	0.30	
Follow-up time*breast developing cancer	0.009 (0.004; 0.014)	< 0.001		0.009 (0.004; 0.014)	< 0.001	
Body mass index (kg/m^2^)	-0.0004 (-0.0009; 0.00007)	0.09		-0.005 (-0.005; -0.004)	< 0.001	
First- or second-degree family history (versus no)	0.010 (0.004; 0.017)	0.003		0.001 (0.004; 0.016)	0.002	
Use of hormone therapy after menopause (ever versus never)	0.002 (-0.002; 0.006)	0.35		-0.001 (-0.005; 0.003)	0.52	
Use of alcohol (ever versus never)	0.023 (0.018; 0.028)	< 0.001		0.015 (0.010; 0.019)	< 0.001	
Smoking (ever versus never)	-0.007 (-0.011; -0.003)	0.001		-0.010 (-0.014; -0.006)	< 0.001	
Constant	2.90 (2.82; 2.97)	< 0.001		2.02 (1.95; 2.08)	< 0.001	
Random effect constant variance	0.042 (0.041; 0.042)	n.a.		0.034 (0.034; 0.035)	n.a	

**Box-Cox transformed

§ Adjusted for age at first screening examination, breast volume, history of benign breast disease, follow-up time, body mass index, family history, hormone therapy, use of alcohol and smoking

& Breasts not developing cancer in women without breast cancer

n.a. – not applicable

## Discussion

In this study, using individual data from 78,182 women, we found that absolute and percent dense volume estimated from screening mammograms decreased to a lower extent in breasts developing cancer compared to the contralateral breasts and breasts in women without breast cancer, over three consecutive screening rounds, plus 2 years of follow-up for interval cancer, a maximum of 6 years of follow-up. We also observed that unadjusted mean absolute dense volume slightly increased in breasts developing interval cancer but was stable in breasts developing screen-detected cancer over three consecutive screening rounds.

High mammographic density presented as high absolute and percent dense volume has previously been linked to an increase in stromal cells and decrease in fat, while the correlation between an increase in epithelial cells and density has been shown to be inconsistent [36, 37]. It is possible that a slower reduction or a slight increase in absolute dense volume in breasts developing cancer in our study could be due to a stable amount or proliferation of stromal components, their signaling activity and substances associated with their growth [37, 38]. The increase in absolute dense volume, specifically in breasts developing interval cancer, might also be associated with the growth of the tumor itself as well as focal breast edema surrounding the tumor [39]. However, our sensitivity analyses did not indicate any association between tumor diameter and density changes, suggesting that non-deterioration and/or growth of the stromal components might be essential in slowing down the decrease in absolute and percent dense volume in breasts developing cancer over the three consecutive screening rounds.

Studies on longitudinal changes in mammographic density and risk of breast cancer have shown varying results [23, 24, 40–44]. A recent nested case-control cohort study of 947 women attending breast screening during up to 10 years in the U.S., showing that volumetric breast density decreased to a lower extent in breasts developing versus not developing breast cancer [23]. In contrast to the study by Jiang et al., our study included three screening rounds and up to 6 years of follow-up, solely postmenopausal predominantly white women with a higher mean age at entry and lower mean BMI. We observed higher percentages of women classified with VDG1 in our study population (11% for women with cancer and 21% for women without cancer) compared to the percentages of women classified with BI-RADS category a in the study by Jiang et al. (6% for cases and 3% for controls). This might have resulted in a lower overall decrease in percent dense volume by age in our study.

A recent meta-analysis using density data for women who developed and did not develop breast cancer stated that an increase in breast density over time was associated with an increased risk of breast cancer, while conversely, a decrease in breast density over time was associated with a reduced risk [19]. We consider our results on differences in changes in absolute and percent dense volume for women who developed and did not develop breast cancer in line with the findings of the meta-analysis.

Longitudinal differences in mammographic density between breasts can be used to map and estimate changes in breast cancer risk over a woman’s lifetime [24, 45]. Our finding on the difference between breasts developing and not developing cancer over time can therefore be considered an important parameter for more precise longitudinal breast cancer risk estimation and screening personalization. One of the advantages of the longitudinal risk prediction is the continuous risk adjustment based on screening results directly associated with the most recent individualized density changes in each breast [23, 45]. Furthermore, in a recent study from Norway, the increase in AI risk scores in breasts developing cancer and the increased difference between the breasts developing and not developing cancer in the same woman was observed over three consecutive screening rounds [46]. Integrating information on longitudinal changes in mammographic density and AI risk scores in a joint model for breast cancer risk estimation could result in high accuracy prediction tools [25, 26]. However, the practical implementation and use of this estimation in relation to the number of mammographic images analyzed, as well as screening interval and supplemental methods of assessment should be investigated in future studies with longer follow-up.

Women with interval cancer have been shown to have a higher mammographic density and thus reduced mammographic sensitivity compared to women with screen-detected cancer [47]. For women developing interval cancer in our study, absolute and percent dense volume was consistently higher, and breast volume lower compared to women developing screen-detected cancer and those without breast cancer over the three consecutive screening rounds. The risk of breast cancer in association with absolute and percent dense volume is known to be modified by breast volume [22, 48, 49]. If absolute dense volume increases, the associated breast cancer risk is less pronounced in women with high versus low breast volume [22, 48]. Breast adipose tissue is part of the microenvironment surrounding the fibroglandular tissue and can be considered an endocrine organ due to secreting adipokines, aromatase, growth factor and other factors that regulate cellular processes in epithelial cells, which might affect fibroglandular tissue [48]. Our finding of consistently lower breast volume combined with higher absolute and percent dense volume in women developing interval versus screen-detected cancer and women without cancer might underline the need to consider low breast volume in combination with high mammographic density as a possible parameter for personalized approaches, including shorter screening intervals and/or other screening tools. In Europe, supplemental screening with digital breast tomosynthesis, MRI or ultrasound has been recommended by EUSOBI for women with extremely dense breasts since 2022 [6].

The strengths of our study are the registry data with high completeness and validity of information on breast cancer diagnosis and data on mammographic density on an individual and breast level. The objective classification of the density represents another strength of the study. However, this study has several limitations. Firstly, follow-up on breast cancer risk factors, including weight and height for BMI calculation and ever use of hormone therapy after menopause, was not performed for each consecutive screening round among the included women. This could have led to imprecision in estimates for these factors. Secondly, the Norwegian female population attending BreastScreen Norway is ethnically and racially homogeneous and predominantly postmenopausal, which might have implied lower mammographic density than in previous studies [19, 23]. Thirdly, self-reported weight and height measures could lead to misclassification; however, results from a validation study of the questionnaire used showed that women consistently and reasonably accurately reported their weight and height [50]. Fourthly, Volpara tends to underestimate percent dense volume in very dense breasts which might have resulted in less accurate estimates and lower magnitude of changes over time for percent dense volume among women with high absolute dense volume and low breast volume [51–53].

## Conclusions

We observed that a longitudinal decrease in absolute and percent dense volume was slower for breasts developing cancer compared to breasts not developing cancer and breasts in women without cancer. These findings imply that longitudinal changes in absolute and percent dense volume might be used for more precise up to 6 years breast cancer risk estimation and thus risk stratifications.

## Electronic supplementary material

Below is the link to the electronic supplementary material.

**Supplementary Material 1**: **Additional file 1Table S1** ABC. Mean values with standard deviation (SD) and median values with interquartile range (IQR) for (A) Absolute dense volume; (B) Breast volume; and (C) Percent dense volume for breasts developing or not developing screen-detected or interval cancer during three consecutive screening rounds of biennial screening in BreastScreen Norway, 2007–2020. **Table S2**. Estimates obtained from a linear-mixed regression model with 95% confidence intervals (CI) showing change in absolute (cm^3^) and percent (%) dense volume on a breast level for 78,182 women screened over three consecutive screening rounds and association with breast cancer in BreastScreen Norway, 2007–2020. **Table S3**. Spearman correlation coefficient between breast volume and body mass index for 66,596 women with available data, screened in BreastScreen Norway, 2007–2020. **Table S4**. Estimates obtained from a linear-mixed regression model with 95% confidence intervals (CI) showing change in mean absolute (cm^3^) and percent (%) dense volume on an individual level for 42,894 women screened over three consecutive screening rounds and association with breast cancer in BreastScreen Norway, 2007–2020.

**Supplementary Material 2**: **Additional file 1 Fig. S1**: Histograms of absolute dense volume and percent dense volume and their corresponding Box-Cox transformed distributions. **Fig. S2**: QQ Plots for the normality of residuals for absolute dense volume and percent dense volume, and their corresponding QQ Plots for the normality of residuals with the improved fit due to Box-Cox transformation.

## Data Availability

No datasets were generated or analysed during the current study.

## Abbreviations

EUSOBI: European society of breast imaging
BI-RADS: Breast imaging and reporting data system
CE: Conformité Européenne
FDA: Food and drug administration
BMI: Body mass index
AI: Artificial intelligence
CC: Craniocaudal
MLO: Mediolateral oblique
VDG: Volpara density grade
DCIS: Ductal carcinoma in situ
SD: Standard deviation
CI: Confidence interval
IQR: Interquartile range
