# Supplementary material for: Mammographic density by time and breast: a retrospective cohort study from BreastScreen Norway
Source: Breast Cancer Res. 2025 May 16;27:83. doi: 10.1186/s13058-025-02037-2 (PMC12083168; doi:10.1186/s13058-025-02037-2)
Supplement: Supplementary file 1 — Supplementary Material 1: Additional file 1Table S1 ABC. Mean values with standard deviation (SD) and median values with interquartile range (IQR) for (A) Absolute dense volume; (B) Breast volume; and (C) Percent dense volume for breasts developing or not developing screen-detected or interval cancer during three consecutive screening rounds of biennial screening in BreastScreen Norway, 2007–2020. Table S2. Estimates obtained from a linear-mixed regression model with 95% confidence intervals (CI) showing change in absolute (cm3) and percent (%) dense volume on a breast level for 78,182 women screened over three consecutive screening rounds and association with breast cancer in BreastScreen Norway, 2007–2020. Table S3. Spearman correlation coefficient between breast volume and body mass index for 66,596 women with available data, screened in BreastScreen Norway, 2007–2020. Table S4. Estimates obtained from a linear-mixed regression model with 95% confidence intervals (CI) showing change in mean absolute (cm3) and percent (%) dense volume on an individual level for 42,894 women screened over three consecutive screening rounds and association with breast cancer in BreastScreen Norway, 2007–2020. [file 13058_2025_2037_MOESM1_ESM.docx]

Additional file 1

Table S1ABC Mean values with standard deviation (SD) and median values with interquartile range (IQR) for A. Absolute dense volume; B. Breast volume; and C. Percent dense volume for breasts developing or not developing screen-detected or interval cancer during three consecutive screening rounds of biennial screening in BreastScreen Norway, 2007-2020

|  | | First screening round | Second screening round | Third screening round |
| --- | --- | --- | --- | --- |
| **A. Absolute dense volume (cm^3^)** | |  |  |  |
| Women developing screen-detected cancer (n=970) | |  |  |  |
| Breast developing cancer | Mean (SD) | 49.6 (24.9) | 49.5 (25.3) | 49.6 (25.2) |
|  | Median (IQR) | 43.0 (32.6 - 60.1) | 43.1 (32.1 - 60.3) | 43.5 (32.4 - 60.2) |
| Breast not developing cancer | Mean (SD) | 49.6 (25.5) | 48.6 (25.2) | 48.9 (25.5) |
|  | Median (IQR) | 43.6 (31.3-60.1) | 41.7 (31.1 - 59.6) | 42.6 (31.2 - 59.7) |
| Women developing interval cancer (n=308) |  |  |  |  |
| Breast developing cancer | Mean (SD) | 54.7 (32.1) | 55.2 (34.2) | 56.2 (32.7) |
|  | Median (IQR) | 45.8 (34.2 - 65.6) | 43.5 (34.0 - 67.5) | 47.4 (34.7 - 67.7) |
| Breast not developing cancer | Mean (SD) | 56.2 (35.9) | 55.7 (36.5) | 54.6 (32.6) |
|  | Median (IQR) | 45.4 (33.6 - 69.3) | 44.1 (33.0 - 65.3) | 43.9 (33.6 - 66.6) |
| Women developing screen-detected or interval cancer (n=1278) | |  |  |  |
| Breast developing cancer | Mean (SD) | 50.8 (26.9) | 50.8 (27.8) | 51.2 (27.5) |
|  | Median (IQR) | 43.5 (32.9 - 61.3) | 43.2 (32.5 - 62.3) | 44.2 (32.9 - 62.2) |
| Breast not developing cancer | Mean (SD) | 51.2 (28.5) | 50.3 (28.5) | 50.1 (27.7) |
|  | Median (IQR) | 44.3 (31.8 - 61.3) | 42.5 (31.8 - 60.8) | 42.7 (31.7 - 60.5) |
| Normal breasts (n=76,904) |  |  |  |  |
| Both breasts | Mean (SD) | 45.0 (24.0) | 44.4 (23.5) | 43.9 (23.2) |
|  | Median (IQR) | 39.1 (29.5 - 53.3) | 38.7 (29.2 - 52.7) | 38.3 (28.9 - 52.2) |
|  |  |  |  |  |
| **B. Breast volume (cm^3^)** |  | First screening round | Second screening round | Third screening round |
| Women developing screen-detected cancer (n=970) | |  |  |  |
| Breast developing cancer | Mean (SD) | 813.4 (419.2) | 831.0 (416.8) | 846.5 (421.8) |
|  | Median (IQR) | 736.6 (507.3 - 1030.5) | 751.4 (533.2 - 1058.1) | 770.8 (540.8 - 1088.3) |
| Breast not developing cancer | Mean (SD) | 813.4 (427.9) | 830.7 (428.7) | 851.4 (433.7) |
|  | Median (IQR) | 724.1 (507.2 - 1038.1) | 742.5 (540.2 - 1036.8) | 779.8 (538.3 - 1101.4) |
| Women developing interval cancer (n=308) | |  |  |  |
| Breast developing cancer | Mean (SD) | 710.9 (387.5) | 731.9 (392.2) | 744.5 (383.6) |
|  | Median (IQR) | 640.4 (422.5 - 907.3) | 666.0 (469.1 - 924.6) | 670.8 (470.5 - 946.3) |
| Breast not developing cancer | Mean (SD) | 709.6 (381.0) | 733.1 (388.8) | 743.4 (385.1) |
|  | Median (IQR) | 629.6 (435.7 - 961.0) | 651.7 (447.3 - 939.2) | 651.9 (473.6 - 973.1) |
| Women developing screen-detected or interval cancer (n=1278) | | |  |  |
| Breast developing cancer | Mean (SD) | 788.7 (414.0) | 807.1 (413.0) | 822.0 (415.0) |
|  | Median (IQR) | 712.6 (489.5 - 1014.2) | 733.8 (512.7 - 1022.8) | 746.9 (524.2 - 1051.6) |
| Breast not developing cancer | Mean (SD) | 788.4 (419.3) | 807.2 (421.3) | 825.3 (424.9) |
|  | Median (IQR) | 703.7 (490.2 - 1012.9) | 721.8 (520.5 - 1019.4) | 739.7 (528.3 - 1050.5) |
| Normal breasts(n=76,904) | |  |  |  |
| Both breasts | Mean (SD) | 798.4 (414.8) | 817.0 (413.3) | 836.9 (415.9) |
|  | Median (IQR) | 729.1 (498.0 - 1028.3) | 748.5 (420.4 - 1045.1) | 773.8 (537.0 - 1068.9) |
|  |  |  |  |  |
| **C. Percent dense volume (%)** |  | First screening round | Second screening round | Third screening round |
| Women developing screen-detected cancer (n=970) | |  |  |  |
| Breast developing cancer | Mean (SD) | 7.2 (4.4) | 7.0 (4.2) | 7.0 (4.2) |
|  | Median (IQR) | 6.0 (4.3 - 8.8) | 5.7 (4.1 - 8.5) | 5.8 (4.1 - 8.6) |
| Breast not developing cancer | Mean (SD) | 7.2 (4.4) | 6.8 (4.0) | 6.9 (4.2) |
|  | Median (IQR) | 5.9 (4.2 - 8.8) | 5.6 (4.1 - 8.3) | 5.6 (4.1 - 8.4) |
| Women developing interval cancer (n=308) | |  |  |  |
| Breast developing cancer | Mean (SD) | 9.3 (5.9) | 8.8 (5.4) | 8.9 (5.4) |
|  | Median (IQR) | 7.2 (5.1 - 11.4) | 7.1 (5.1 - 11.1) | 7.3 (5.1 - 11.1) |
| Breast not developing cancer | Mean (SD) | 9.3 (5.8) | 8.7 (5.3) | 8.8 (5.3) |
|  | Median (IQR) | 7.5 (5.2 - 11.0) | 7.3 (5.1 - 10.6) | 7.4 (5.1 - 10.8) |
| Women developing screen-detected or interval cancer (n=1278) | | |  |  |
| Breast developing cancer | Mean (SD) | 7.7 (4.9) | 7.4 (4.6) | 7.3 (4.3) |
|  | Median (IQR) | 6.2 (4.4 - 9.4) | 5.9 (4.3 - 9.0) | 6.0 (4.2 - 9.1) |
| Breast not developing cancer | Mean (SD) | 7.7 (4.9) | 7.3 (4.3) | 7.1 (4.3) |
|  | Median (IQR) | 6.2 (4.5 - 9.6) | 5.9 (4.1 - 8.7) | 5.9 (4.1 - 8.7) |
| Normal breasts (n=76,904) | |  |  |  |
| Both breasts | Mean (SD) | 6.8 (4.4) | 6.5 (4.1) | 6.3 (4.1) |
|  | Median (IQR) | 5.4 (3.9 - 8.2) | 5.2 (3.7 - 7.8) | 4.9 (3.5 - 7.7) |

Table S2. Estimates obtained from a linear-mixed regression model with 95% confidence intervals (CI) showing change in absolute (cm^3^) and percent (%) dense volume on a breast level for 78,182 women screened over three consecutive screening rounds and association with breast cancer in BreastScreen Norway, 2007-2020

|  | **Absolute dense volume (cm^3^) **** | |  | **Percent dense volume (%) **** | |
| --- | --- | --- | --- | --- | --- |
|  | n=234,546 examinations  (n=78,182 women) ^§^ | |  | n=234,546 examinations  (n=78,182 women) ^§^ | |
| Variable | **Estimate (95% CI)** | **P-value** |  | **Estimate (95% CI)** | **P-value** |
| Age at first screening examination (years) | -0.007 (-0.008; -0.0062) | <0.001 |  | -0.0073 (-0.008; -0.006) | <0.001 |
| Breast volume (cm^3^) | 0.0002 (0.0002; 0.0002) | <0.001 |  | -0.0004 (-0.0004; -0.0004) | <0.001 |
| Benign breast disease (ever) | -0.0004 (-0.010; 0.009) | 0.94 |  | 0.007 (-0.002; 0.016) | 0.14 |
| Follow-up time (screening round) | -0.011 (-0.011; -0.010) | <0.001 |  | -0.014 (-0.014; -0.013) | <0.001 |
| Breast not developing cancer | 0.063 (0.049; 0.077) | <0.001 |  | 0.059 (0.046; 0.072) | <0.001 |
| Breast developing cancer | 0.055 (0.041; 0.069) | <0.001 |  | 0.050 (0.037; 0.063) | <0.001 |
| Follow-up time*breast not developing cancer | 0.001 (-0.003; 0.004) | 0.78 |  | 0.002 (-0.002; 0.006) | 0.31 |
| Follow-up time*breast developing cancer | 0.009 (0.005; 0.0013) | 0.01 |  | 0.010 (0.006; 0.013) | 0.01 |
| Constant | 2.97 (2.91; 3.02) | <0.001 |  | 1.94 (1.89; 1.99) | <0.001 |

**Box-Cox transformed

^§^ Adjusted for age at first screening examination, breast volume, history of benign breast disease, and follow-up time

Table S3. Spearman correlation coefficient between breast volume and body mass index for 66,596 women with available data, screened in BreastScreen Norway, 2007-2020

|  | Body mass index (kg/m^2^) | Breast volume (cm^3^) |
| --- | --- | --- |
| Body mass index (kg/m^2^) | 1.0 |  |
| Breast volume (cm^3^) | 0.6387 | 1.0 |

Table S4. Estimates obtained from a linear-mixed regression model with 95% confidence intervals (CI) showing change in mean absolute (cm^3^) and percent (%) dense volume on an individual level for 42,894 women screened over three consecutive screening rounds and association with breast cancer in BreastScreen Norway, 2007-2020

|  | **Absolute dense volume (cm^3^) **** | |  | **Percent dense volume (%) **** | |
| --- | --- | --- | --- | --- | --- |
|  | n=128,682 examinations  (n=42,894 women)^§^ | |  | n=128,682 examinations  (n=42,894 women)^§^ | |
| Variable | **Estimate (95% CI)** | **P-value** |  | **Estimate (95% CI)** | **P-value** |
| Age at first screening examination (years) | -0.006 (-0.008; -0.005) | <0.001 |  | -0.006 (-0.008; -0.005) | <0.001 |
| Breast volume (cm^3^) | 0.0002 (0.0002; 0.0002) | <0.001 |  | -0.0004 (0.0004; 0.0004) | <0.001 |
| Benign breast disease (ever) | 0.004 (-0.010; 0.017) | 0.596 |  | 0.009 (-0.010; 0.017) | 0.128 |
| Follow-up time (screening round) | -0.009 (-0.010; -0.008) | <0.001 |  | -0.014 (-0.015; -0.014) | <0.001 |
| Women developing cancer | 0.056 (0.035; 0.075) | <0.001 |  | 0.060 (0.42; 0.077) | <0.001 |
| Follow-up time*woman developing cancer | 0.006 (0.001; 0.011) | 0.027 |  | 0.007 (0.002; 0.012) | 0.009 |
| Body mass index (kg/m^2^) | 0.001 (0.000; 0.002) | 0.001 |  | -0.003 (-0.003; -0.002) | <0.001 |
| First- or second-degree family history | 0.010 (0.003; 0.017) | 0.005 |  | 0.009 (0.003; 0.015) | 0.003 |
| Ever use of hormone therapy | 0.002 (-0.002; 0.007) | 0.265 |  | -0.001 (-0.005; 0.003) | 0.487 |
| Ever use of alcohol | 0.024 (0.019; 0.029) | <0.001 |  | 0.012 (0.008; 0.017) | <0.001 |
| Ever smoking | -0.006 (-0.010; -0.002) | 0.005 |  | -0.010 (-0.014; -0.006) | <0.001 |
| Constant | 2.880 (2.800;2.959) | <0.001 |  | 1.982 (1.901; 2.043) | <0.001 |
| Constant | 0.042 (0.042; 0.043) |  |  | 0.034 (0.033; 0.034) |  |

**Box-Cox transformed

^§^ Adjusted for age at first screening examination, breast volume, history of benign breast disease, follow-up time, body mass index, family history, hormone therapy, use of alcohol and smoking
