# Supplementary material for: Mammographic density by time and breast: a retrospective cohort study from BreastScreen Norway
Source: Breast Cancer Res. 2025 May 16;27:83. doi: 10.1186/s13058-025-02037-2 (PMC12083168; doi:10.1186/s13058-025-02037-2)

Additional file 2

Figure S1 Histograms of absolute dense volume and percent dense volume and their corresponding Box-Cox transformed distributions


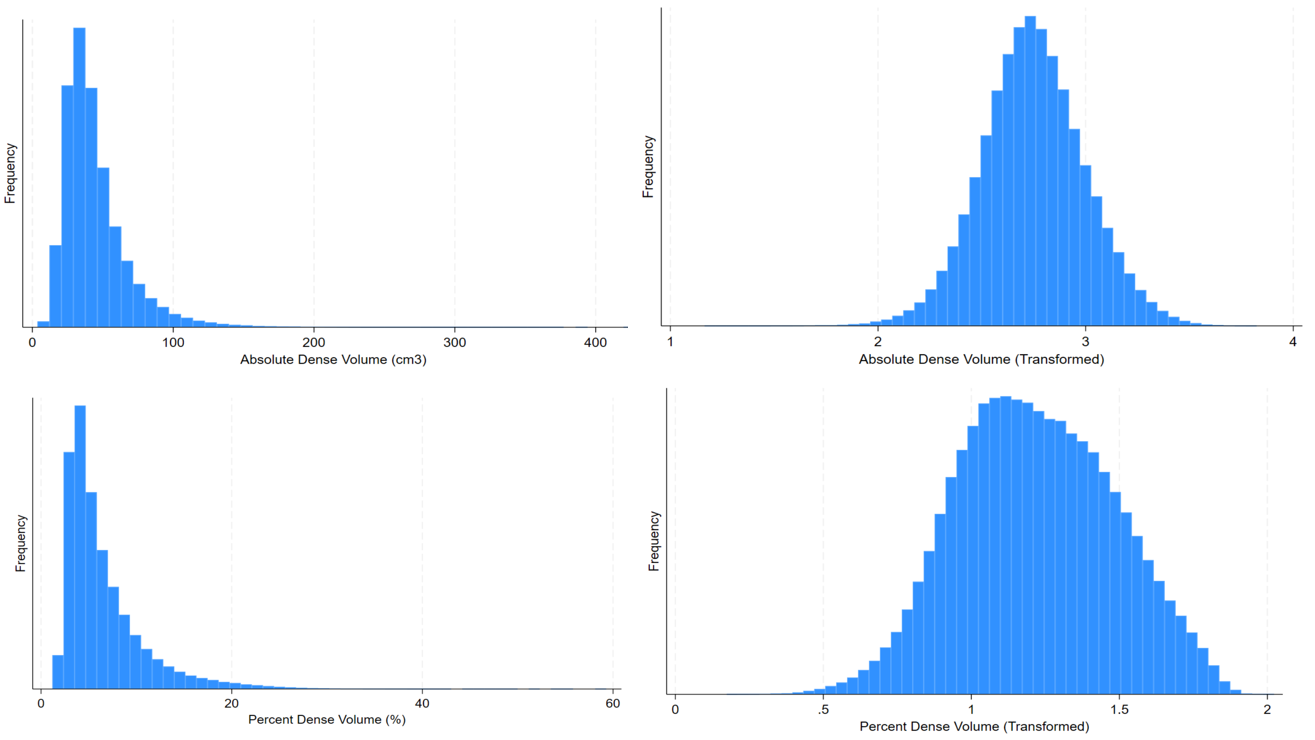


Figure S2. QQ Plots for the normality of residuals for absolute dense volume and percent dense volume, and their corresponding QQ Plots for the normality of residuals with the improved fit due to Box-Cox transformation


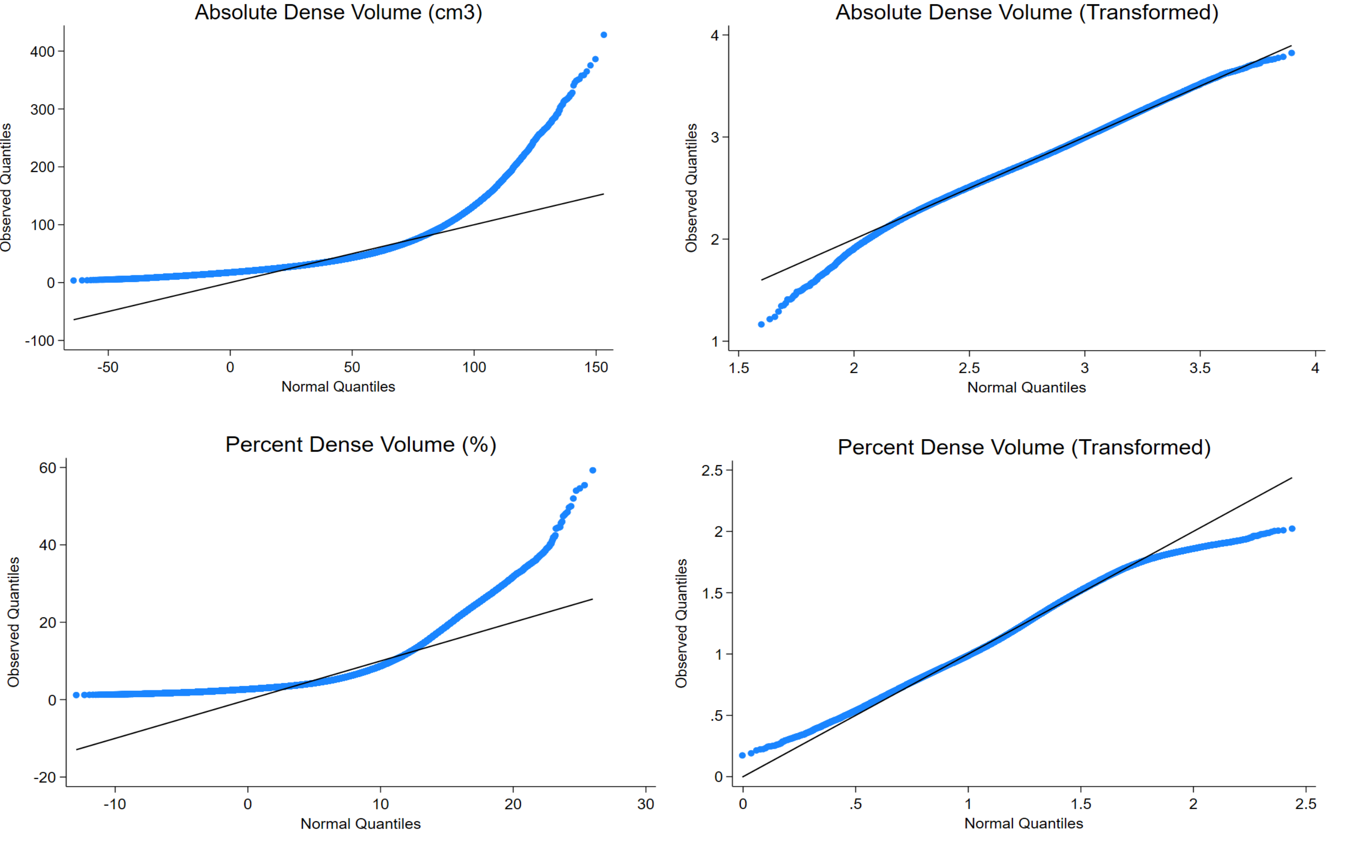

Supplement: Supplementary file 2 — Supplementary Material 2: Additional file 1 Fig. S1: Histograms of absolute dense volume and percent dense volume and their corresponding Box-Cox transformed distributions. Fig. S2: QQ Plots for the normality of residuals for absolute dense volume and percent dense volume, and their corresponding QQ Plots for the normality of residuals with the improved fit due to Box-Cox transformation. [file 13058_2025_2037_MOESM2_ESM.docx]
